# Stakeholder perspectives on the WHO Mental Health Gap Action Programme Intervention Guide (mhGAP-IG) in humanitarian settings: a qualitative study in Lebanon and Iraq

**DOI:** 10.3389/frhs.2026.1726804

**Published:** 2026-03-17

**Authors:** Marcello Roriz de Queiroz, Martina Valente, Ives Hubloue, Francesco Della Corte

**Affiliations:** 1 Center for Research and Training in Disaster Medicine, Humanitarian Aid and Global Health (CRIMEDIM), University of Eastern Piedmont, Novara, Italy; 2 Research Group on Emergency and Disaster Medicine (ReGEDIM), Vrije Universiteit Brussel, Brussel, Belgium; 3 Department for Sustainable Development and Ecological Transition, Università del Piemonte Orientale, Vercelli, Italy

**Keywords:** humanitarian settings, implementation, mental health, mental health gap action programme intervention guide, primary health care

## Abstract

**Background:**

The mental health treatment gap remains substantial in countries affected by humanitarian crises, where fragile health systems and structural instability hinder service delivery. The World Health Organization's Mental Health Gap Action Programme (mhGAP) aims to scale up evidence-based mental health care through integration into primary health care. However, its real-world implementation in humanitarian settings remains underexplored.

**Methods:**

We conducted a qualitative study using in-depth individual interviews with 12 stakeholders, including primary care practitioners, mhGAP trainers, field supervisors and health managers involved in the implementation of WHO mhGAP training over the past five years in Lebanon and Iraq. Thematic analysis was applied to explore perceptions of training effectiveness, implementation dynamics, and contextual determinants influencing the mental health service delivery at primary health care level.

**Results:**

The main findings of the study revolved around the following dimensions: (1) cases involving suicide risk and substance abuse are considered more challenging, requiring additional skills and coordination with specialized services; (2) the stigma surrounding mental health within communities and among healthcare providers further hinders access to care; (3) poor continuity of care restrict effective mental health care delivery; (4) the mhGAP training enhances knowledge and skills, but changing attitudes and clinical practices are directly influenced by local health systems and policies; (5) continuous supervision is crucial for changing clinical practices and ensuring care aligns with evidence-based guidelines; (6) effective monitoring mechanisms are needed to ensure the training meets its objectives and improves access to mental health care at the primary level.

**Conclusions:**

The mhGAP training experiences have shown potential in bridging the mental health treatment gap, while a stark disparity in access to mental health services is evident within countries, which is particularly acute for populations in humanitarian settings where instability and resource scarcity compound mental health challenges. This accentuates the urgency of targeting investments in the most underserved areas, and calling for a nuanced approach that recognizes the complex interplay of social, cultural and economic factors influencing health service delivery.

## 1 Introduction

There is a consensus among healthcare professionals and global health specialists that the integration of mental health care into primary health care levels represents a shift in the global approach to addressing the growing need for mental health services (1). This consensus is based on the principles of accessibility, cost-effectiveness, and continuity of mental health care (2, 3). Despite this agreement, the treatment gap for mental health remains a significant challenge. According to the World Health Organization (4, 51), treatment coverage for mental disorders varies substantially across countries, regions, and from one mental disorder to another. High-income countries report treating approximately 71% of individuals with psychotic disorders, whereas in low-income countries only about 12% of people with these conditions receive mental health care (5). Coverage for depression also remains particularly limited: minimum adequate treatment reaches 27% of cases in high-income countries but is considerably lower in low-and-middle-income countries (LMICs), frequently falling below 3% in parts of sub-Saharan Africa and Asia (6).

During humanitarian crises, the integration of mental health care at the primary health level is particularly challenging, but extremely necessary. Humanitarian crises, often triggered or exacerbated by armed conflict, force people to leave their homes in extremely difficult conditions and to live without access to the most essential items. In such contexts, the prevalence and burden of common mental disorders, including mild forms of depression, anxiety, and post-traumatic stress disorder, are significantly higher when compared to global estimates (7).

The prevalence of mental disorders is higher in Middle Eastern countries than in global figures (8, 9), which is commonly associated with ongoing conflicts and humanitarian crises in the region (10). Almost 60 million people across the Middle East region need humanitarian assistance at different levels, and local health systems face dramatic challenges in scaling up mental health services to deliver effective care to the most vulnerable groups (11, 20).

### 1.1 The mental health gap action programme (mhGAP)

As part of global efforts to address the large burden of mental health conditions, national health authorities guided by the WHO and international organizations have implemented initiatives for scaling up care for mental health and substance use disorders at the primary health level. In 2008, the WHO launched the Mental Health Gap Action Programme (mhGAP) aiming to scale up care for mental, neurological, and substance use (MNS) disorders in non-specialized health care settings, which includes the mhGAP Intervention Guide (mhGAP-IG), a guidance package on the diagnosis and management of MNS disorders (12). The revised *mhGAP-IG 2.0* and the *mhGAP Humanitarian Intervention Guide* (HIG) versions were also released in 2015 (52).

Since then, mhGAP training packages have been extensively disseminated across countries worldwide. Studies have demonstrated the benefits of the use of mhGAP-IG in training for improving the knowledge, attitudes, and confidence of health workers in relation to mental health disorders (14), particularly in enhancing the engagement of primary care practitioners with mental health.

Nevertheless, evidence shows that most of the mhGAP experiences have either never been evaluated or have not been shared as widely as they could be. Considering this reality, Spagnolo & Lal (15) conducted an extensive review of the grey literature and identified more than 90 countries in which mhGAP-IG was implemented.

While it is notable that most mhGAP initiatives are implemented in LMICs, many of which facing humanitarian crises, countries of the Eastern Mediterranean Region (16), which includes all thirteen Arabic countries of the Middle East (17), represent only 11.49% of the initiatives reported (15). During an updated systematic review of the literature on mhGAP-IG, Keynejad (18) and colleagues confirmed that despite the dramatic increase in published studies on mhGAP-IG use and evaluation, notably few studies (7%) have been conducted in the Eastern Mediterranean region.

The mhGAP-IG has been used as a key training tool in humanitarian crises; however, its effectiveness remains under-evaluated in the peer-reviewed literature. Most existing studies focus on acquired knowledge among primary health care (PHC) providers, with limited evidence linking mhGAP training to improved access to mental health care at the primary care level (19).

Lebanon faces a challenging context for integrating mental health into primary health care. According to the United Nations High Commission for Refugees, the country hosts more than 1.5 million Syrians displaced by the conflict (50), representing the highest proportion of refugees to host population worldwide. This demographic pressure has exacerbated already high and growing rates of non-communicable diseases (NCDs) and mental health conditions, affecting both the Lebanese population and the refugee community, and underscoring the urgent need for stronger primary health care services. In addition to the Syrian crisis, Lebanon has been experiencing an unprecedented economic and financial crisis, further exacerbated by the COVID-19 pandemic and the Beirut port explosion. These compounded shocks have significantly weakened the health system, limiting state capacity to ensure equitable access to health care services for the general population (21). Within this fragile system, initiatives such as the WHO mhGAP play a central role in expanding access to mental health care (22), aligned with the efforts made by the National Mental Health Program (NMHP) in Lebanon in collaboration with humanitarian agencies (23, 24).

In Iraq, mental health has remained a highly challenging issue to address after two decades shaped by recurrent wars, protracted conflicts, and ongoing political instability (25). Modern psychiatric services in Iraq were originally established in the 1930s, marking one of the earliest institutional mental health developments in the region; however, decades of sanctions and instability have repeatedly disrupted service continuity and system development (26).

The cumulative effects of these crises have deeply scarred the population, where fear and trauma have become widespread. Despite the magnitude of these problems, research and programmatic interventions have been limited, leaving significant gaps in knowledge regarding their epidemiology (27). Moreover, the destruction of institutional archives and documentation during successive conflicts has resulted in important gaps and inconsistencies in official health data, limiting the reliability of historical service assessments (26, 28).

The mental health system remains limited, and service provision is centralized in a small number of specialized psychiatric hospitals, while community mental health centers are largely absent (29). Mental health professionals face multiple difficulties, including limited training opportunities, scarce resources, and persistent stigma surrounding mental health care (30). Epidemiological estimates suggest that nearly one in five Iraqis will experience a mental health condition over their lifetime, with anxiety disorders, post-traumatic stress disorder (PTSD), and depression being the most common (31). Recognizing these challenges, the Ministry of Health initiated efforts in the late 2000s to rebuild and decentralize mental health services, notably through integrating mental health care into primary health care (26).

### 1.2 Study objectives

The objective of this study is to evaluate the perceptions and understanding of key actors (primary health practitioners and health supervisors) regarding the effects of mhGAP training initiatives on improving the quality and access to mental health care at the primary level in their respective contexts. To the best of our knowledge, there are few in-depth qualitative studies on the implementation of mhGAP-IGs in countries affected by humanitarian crises. Capturing the lived experiences of primary care practitioners, mhGAP trainers, supervisors, as well as local mental health managers in Lebanon and Iraq can contribute to a better understanding of the strengths and challenges of mhGAP implementation and, overall, to a discussion of the real-world barriers and necessary measures to integrate mental health into primary health care in humanitarian settings.

## 2 Methods

### 2.1 Study design

An in-depth qualitative study was conducted using semi-structured interviews to explore the perceptions of key stakeholders regarding the effectiveness of the mhGAP training package at the primary health care level in Lebanon and Iraq.

Following the criterion of prioritizing countries in the Middle East facing humanitarian crises and with an established background of mhGAP implementation at the national level, Lebanon and Iraq were purposively selected, as local partners ensured ethical clearance and feasibility for in-depth qualitative interviewing. The initial study design also included Syria as a third country; however, access to primary care practitioners and health managers was not granted.

Interviews were conducted until thematic saturation was reached, defined as the point at which no new codes or concepts emerged in two consecutive interviews, a process verified through team discussions during the coding phase (32, 33).

The in-depth individual interviews were deemed appropriate for exploring participants’ views and perceptions of training interventions, including their perspectives on the contextual challenges and dimensions of effectiveness. The interviews were conducted by a researcher with previous experience in qualitative research and a background in global mental health, including field experience in primary health care, as a trainer and mental health supervisor.

### 2.2 Sampling strategy

Purposive sampling was used to select and recruit interviewees. Three main criteria were considered for the selection of interviewees: (a) location: health personnel working in regions affected by humanitarian crises in Lebanon and Iraq; (b) profile: primary health care practitioners, field supervisors, and health managers at the local or national level (e.g., Ministry of Health); and (c) mhGAP training experience: stakeholders who have been involved in the implementation of *WHO* mhGAP-IG over the past five years. Stakeholders were contacted via email. An information leaflet clarifying the scope of the study, including its objectives, methodology, and information on data analysis and ethical implications, was sent by email. Upon confirmation of participation, stakeholders were offered several time slots for the interviews at their convenience.

### 2.3 Data collection

A semi-structured interview guide with leading and probing questions was developed while keeping in mind the objective of the study (Annex). The interview guide focused on four thematic aspects: (1) mental health needs and service gaps encountered at the primary health level, (2) perceptions of the mhGAP training experience, (3) perceived effects of the mhGAP training, and (4) challenges and recommendations for mhGAP implementation. Specific attention was given to the main barriers and facilitators encountered by stakeholders to improve access to mental health care at the primary health level. The guide was piloted with a mental health specialist and a general practitioner, and it was revised following their feedback. The same interview guide was used to interview all respondents, with minor linguistic adaptations to address stakeholders with different roles (practitioners, field supervisors, and health managers). The interviews were conducted from May to July 2023 and lasted from 60 to 80 min each. The interviews were conducted using *Zoom* and *Microsoft Teams*, and upon consent, the interviews were recorded. Notes were taken during the interviews.

Whenever possible, interviews were conducted in English. Four interviews in Iraq required the deployment of an Arabic-speaking assistant, a local physician with practical experience in mhGAP-IG. The transcripts were subsequently translated into English through a double translation process for analysis.

### 2.4 Data analysis and reporting

The interviews were transcribed verbatim using *Sonix* software. The transcripts were manually checked for completeness and quality. A code guide was developed based on the study objectives, the concepts described by the *WHO* mhGAP framework, and the dimensions of mental health training effectiveness explored by previous studies and described in the literature. The code guide was used to deductively code the transcripts, and it was flexible enough to be adapted if new codes emerged inductively from the text. The data were coded using Atlas.ti and analysed following a thematic analysis approach (34). The results were reported following the Standards for Reporting Qualitative Research- SRQR (35). A completed SRQR checklist is available in Supplementary Material 1.

### 2.5 Ethics declarations

Upon consent to participate, a digital copy of the informed consent form was sent to all interviewees a few days before the interviews. Interviewees were fully informed of their rights, including voluntary participation and the option to withdraw consent without consequences. The interviewees had the opportunity to ask questions and seek clarification before the interviews. The study was approved by the Ethics Committee of the A.O.U. “Maggiore della Carità” (Protocol 699/CE Study number CE 088/2023), and by the Institutional Review Board Rafik Hariri University Hospital in Lebanon (RHUH 17.2023). The data collected were anonymized, and access to the data was restricted to the coauthors of this paper only. The study included health personnel only, and it did not involve patients or vulnerable groups.

## 3 Results

### 3.1 Characteristics of the sample

In total, 12 key stakeholders were enrolled in this study (Table 1). Of the 12 participants, seven were males and five were females. Four participants were primary care practitioners, and two had less than five years of experience at the primary health level. Five participants played a role as trainer or supervisor of the *WHO* mhGap training in Lebanon and Iraq over the last five years. Two participants had managerial roles, overseeing the mental health training initiatives at the respective Ministry of Health. One participant had been working as an international researcher with experience in evaluating the effectiveness of mhGAP training^1^.

**Figure 1:**
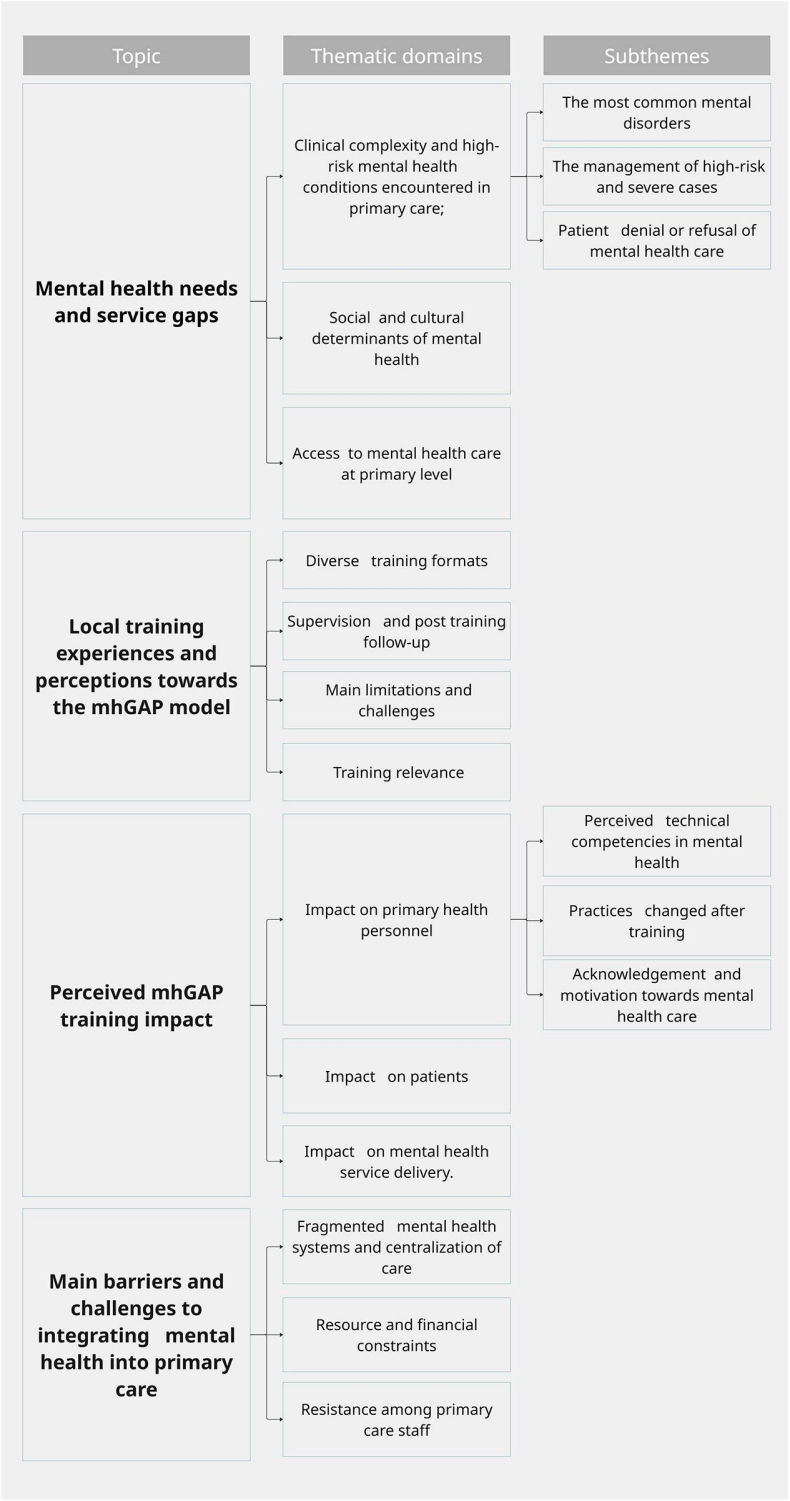
Summary of findings.

The Figure 1 presents a synthesis of the main findings emerging from the thematic analysis, organized into four thematic domains and their corresponding subthemes The domains include: (1) mental health needs and service gaps; (2) local training experiences and perceptions towards the mhGAP model; (3) perceived mhGAP training impact; and (4) main barriers and challenges to integrating mental health into primary care.

**Table 1:** Characteristics of the interviewees.

Country	Role
Lebanon	Health Manager
	Primary care practitioner
	Primary care practitioner
	mhGAP trainer and supervisor
	Mental health supervisor
Iraq	Health Manager
	Primary care practitioner
	Primary care practitioner
	Mental health supervisor
	mhGAP trainer
	Primary care supervisor
External- International organization^a^	International researcher

^a^ International researcher of mental health systems with previous experience in both countries.

### 3.2 Mental health needs and service gaps

The following thematic domains emerged when examining the most common mental needs seen in primary care, alongside the availability of local health services and the main gaps in providing stepped care for mental health: (1) clinical complexity and high-risk mental health conditions encountered in primary care; (2) social and cultural determinants of mental health; and (3) access to mental health care at primary level.

#### 3.2.1 Clinical complexity and high-risk mental health conditions encountered in primary care

Within this theme, three interrelated subthemes emerged: (i) the prevalence of common mental disorders, (ii) the management of high-risk and severe cases, and (iii) patient denial or refusal of mental health care. The interviewees identified symptoms of depression and anxiety as the most common mental health conditions encountered in their respective primary care settings. The spectrum of symptoms reported by primary care practitioners varies widely, encompassing persistent low mood, fatigue, and changes in sleep patterns. While some patients may present with mild symptoms, others manifest acute conditions requiring immediate attention. Symptoms of posttraumatic stress disorder (PTSD) were also emphasized by primary care practitioners as a prevalent issue, especially in areas affected by armed conflicts and other emergencies: “*the majority of cases that I see, the mental health cases, are depression..then the anxiety, then PTSD, because since the explosion of August 4th, 2020, we are seeing many cases with PTSD” (*primary care practitioner, Lebanon*).* The escalating cases of substance use were also highlighted by the interviewees, that emphasized the high vulnerability of those patients and the complexity of establishing proper care.

Among the most challenging conditions reported by primary care practitioners are depression coupled with suicidal ideation, especially in assessing suicide risk and defining a follow-up care plan: “*I saw many patients with suicidal ideations and recent suicidal attempts. They came and they already have tried to cut their wrists, or they took pills like a week or a month ago. So it's really risky…when see such patient, I prefer to refer them to the psychiatrist… so this is the main challenging issue to me” (*primary care practitioner, Lebanon).

Although less common, severe mental health conditions are reported by interviewees as complex cases for the primary care practitioners to address. In all the contexts covered by this study, the participants reported that primary care practitioners are not advised to manage themselves cases of severe mental disorders (i.e., schizophrenia or any psychosis) but rather to make the first assessment and refer to psychiatrists when available.

Another challenge reported by the primary health care practitioners are the patients that deny the need of mental health care or refuse to seek professional assistance despite exhibiting clear signs of mental health issues.

#### 3.2.2 Social and cultural determinants of mental health

Interviewees pointed out the interplay of socioeconomic determinants and cultural dynamics that significantly influences the prevalence of mental health issues within individuals and communities. In Lebanon, one noteworthy factor is the economic crisis, which, according to primary care practitioners, has emerged as a major catalyst for the rise of mental health issues. The economic downturn in the country has resulted in job losses, financial instability and increased stress levels, all of which are precipitating and exacerbating mental health conditions: “*the number of people with diabetes mellitus and hypertension increase…and with the depressive disorder increase..a very bad economic situation that our people live*” (primary care practitioner, Lebanon). The economic crisis also prevents primary health care providers from managing mental health problems effectively, such as patients with difficulties attending consultations because of transportation costs.

In Iraq, the drastic economic downturn in the 1990s was also remembered by interviewees: “*In the 90s, the economic restrictions that Iraq faced led to, according to the records, I think 1 million children to permanent disability or death*” (mhGAP trainer, Iraq).

The interviewees mentioned that political instability represents another critical social determinant in the region that acts as a significant stressor for mental health. The uncertainty, fear, and upheaval associated with security issues and political instability can lead to heightened levels of stress and anxiety within communities.

Additionally, intercommunity dynamics, including social stigma towards mental health, influence individuals’ willingness to seek help. The interviewees mentioned the high stigma associated with mental health, which affects both the disclosure of symptoms and the acceptance of mental health interventions*: “In humanitarian settings, especially in the Middle East, the stigma for mental health is still very high. So most of the time, if you find a person with a mental health problem and that person is known in the community, they would say the person is “majnun”, it is crazy, the person is crazy*..*the family tries to hide the person if it has the tendency to go out and shout around in the streets*” (mental health researcher). According to the interviewees, the stigma surrounding mental health is deeply rooted in cultural, religious, and societal norms, contributing to a pervasive reluctance to openly discuss and address mental health issues. The interviewees pointed out the stigma not only from the community but also among health workers: “*you can find large percentages of health staff who are quite skeptical about mental health care because it wasn't part of their education*.” (mental health researcher).

#### 3.2.3 Access to mental health care at primary level

Access to mental health care at the primary level is described as a combination of challenges, especially in areas affected by humanitarian crises. While primary health care and specialized mental health services are apparently accessible without financial hardship (reduced service fees), there often exists a stark disparity in access within the same country. Remote or conflict-affected areas may face limited resources, infrastructure and trained personnel, leading to a lack of mental health services offered in these regions: “*We have centers who have mental health services free of charge..but we have a lot of challenges because during the last years a lot of centers closed because of the shortage of financial funds”* (mhGAP trainer, Lebanon).

Additionally, there is a pervasive issue of mistrust among patients toward the skills of primary health workers in providing mental health care. This skepticism stems from the perception that mental health issues require specialized expertise beyond what is available in general health settings. Moreover, this perception is reinforced by a centralized psychiatric hospital-based model, which is still prevalent in many contexts in the Middle East: “*the huge, the huge problem in this context is that it's somehow centralized, it is hospital based. Sometimes the health system is quite complicated to facilitate the referral from primary health to mental health..*” (mhGAP trainer, Iraq).

Compounding the challenge is the lack of awareness about mental health and the tendency of individuals to seek care at primary health facilities for physical health only. The stigma surrounding mental health may further discourage individuals from explicitly seeking mental health care, leading to delayed or inadequate interventions. As a result, access to mental health care in both primary health settings and specialized services is far from what would be required to address mental health needs: “*I do think there's a very large amount of people who would be in need, but they do not reach the primary care level”* (mental health researcher).

### 3.3 Local training experiences and perceptions of the mhGAP model

The following themes emerged when examining the interviewees’ perceptions of mhGAP training in their respective contexts: (1) diverse training formats, (2) supervision and post training follow-up, (3) main limitations and challenges and (4) training relevance.

#### 3.3.1 Diverse training format

mhGAP training has been implemented in Lebanon and Iraq since the first version was released in 2008. The implementation is normally undertaken both by local Ministries of Health (with or without specific international funding) or implemented directly by international humanitarian organizations. All the training experiences mentioned by the interviewees were adapted from the *WHO* mhGAP manuals (versions 1.0 and 2.0). The training format varied, with selected modules conducted as intensive 4–5 days training or divided into separate modules and sessions with breaks allowing participants to apply their learning between sessions.

The interviewees agreed that the training framework prioritized the modules of depression and essential care and practice: “*I usually stress a lot on the introduction part.I think this is the most important part if you want to start working in this field, talking about the communication, the general principles of care. This part, I find it really very, very interesting”* (mhGAP trainer, Lebanon). The focus on essential care aspects also allows the trainer to further understand the trainees’ expectations and concerns in relation to mental health: “*sometimes they do not want to listen (the patients) because they do not know what to say or what to do..there are some specific contents that could help them to know what they could say in these difficult situations*” (mental health supervisor, Lebanon).

In some contexts, specific training modules were designed for physicians and nurses, while maintaining common content for all participants. The interviews mentioned that all training was conducted by a local psychiatrist or an international expert, depending on the context. In all the reported experiences, the strategy of training local specialists from the Ministries of Health through *training of trainers* was the key approach taken by local authorities to scale up the training initiatives and to adapt them to their local priorities.

Some interviewees mentioned that the training also included presentations about local mental health services, emphasizing guidance on screening, protocols for assessment (i.e., Lebanon), patient follow-up and referral pathways, reflecting the particularities of the local healthcare system.

#### 3.3.2 Supervision and post training follow-up

There is a consensus among the interviewees that a crucial component of mhGAP effectiveness lies in the regular supervision and post-training follow-up of its trainees, which ensures the proper implementation and integration of mental health services into primary healthcare. The interviewees agreed that supervision plays a pivotal role in reinforcing the training content, addressing misconceptions, and providing continuous support: “*I think the supervision meetings are also as important as the trainings because trainings alone are not enough to see if the doctor already got the point or not (*primary care practitioner, Lebanon*).* The mhGAP trainers mentioned that supervision sessions are also important for overcoming trainees’ resistance: “*The training is quite new for the primary health care physicians or nurses. And what I see in my experience is that the first few weeks they are still hesitant to practice it in their settings because they do not feel confident. So, what I was always doing is that we have weekly supervision group sessions for at least the first six weeks”* (mhGAP trainer, Iraq).

The interviewees mentioned that regular supervision in mhGAP training may involve various formats, each with its pros and cons. Regular group supervision sessions, for instance, allow trainees to share experiences, learn from peers, and receive collective guidance from a supervisor but may not address individual-specific learning needs or challenges. On the other hand, onsite one-to-one supervision provides tailored guidance, addressing specific challenges faced by individual trainees. However, this format can be time consuming and might not offer the exchange of experiences and peer technical support found in group sessions:.*we used to visit every doctor in the center where he works, sit with him and see the patients with him, all the patients that are coming and see if he's being able to screen, diagnose and manage the patients. It has a lot of advantages…the negative point of such type of supervision is the time, we used to do 4 or 5 h of driving to sit two hours, two hours and a half with the doctor. So, it takes a lot of time”* (mhGAP trainer, Lebanon).

The framework for post-training follow-up and supervision can also vary significantly among countries, depending on funding and the organization responsible for the implementation: “*Sometimes the supervision is three months, sometimes six months, and I have been doing supervision for more than two years now. So it depends a little bit about the project*” (mhGAP trainer, Lebanon). Interviewees mentioned that communication tools, such as WhatsApp, are widely used to connect trained primary care staff with supervisors, ensuring ongoing support.

There is consensus among interviewees that without regular supervision and follow-up, there is a significant risk that the mhGAP objectives will not be effectively implemented. Supervision ensures that the skills and knowledge acquired during training are correctly and consistently applied, providing a platform for addressing challenges and barriers in real time and ensuring that mental health services are integrated into primary health settings: “*without supervision, there is no implementation”* (mhGAP trainer, Iraq).

#### 3.3.3 Main limitations and challenges

Several limitations and challenges were reported by interviewees in relation to the implementation of mhGAP training in their respective contexts. A notable concern was the short duration of training: “*there is stressed time, and we got very concentrated topics and materials. So I believe it is very useful, but it was very concentrated and very stressed. So, there is need for expanding.*(mental health supervisor, Iraq). A longer period of training is critical, particularly for trainees without prior mental health training: “*We need more people to be trained, also for a longer period..because there are many new topics that we hear for the first time* (primary care practitioner, Iraq).

The lack of continuity in training, due to various regional issues and operational constraints, was also mentioned. The interviewees mentioned that there is a significant dependency on international organizations to develop and sustain training initiatives in both financial and technical aspects. Therefore, the length and selection of training modules often depend on the operational perspectives and funding of the implementing international organizations, which may lead to discontinuity, lack of consistency, and, very often, complicated coordination among local stakeholders.

In some contexts, there is a recognized focus on the screening and referral approach and the need to improve the psychosocial and psychological interventions applicable for primary care within the training modules: “*I do not see… entering into this kind of discussion as well. How to provide psychosocial support, always focusing on identifying and treating by providing psychotropics.*(mental health supervisor, Lebanon). The lack of training continuity, including limited post-training follow-up, can also lead to the risk of potentially harmful prescriptions: “*I do think the technical knowledge is there, but because there's very few case discussions and very limited supervision, what often would happen is if you just follow these flow charts, this may lead to overmedication…* ‘*I've seen lots of cases who were medicated from the first visit without being thoroughly assessed or diagnosed first”* (mental health researcher).

Some interviewees mentioned that on more than one occasion, the psychiatrists recruited as trainers were not familiar with the primary health care model and mhGAP concept and modules, leading to confusion among trainees: “*One of the criteria to be a trainer on mhGAP is to have mental health experience, preferably a psychiatrist. In the context where I worked, most of the psychiatrists are hospital based..this affects the quality of the training because they will keep referring mainly to the hospital management model, which will not fit in primary health care.*” (mhGAP trainer, Iraq).

Finally, some interviews raised the concern of the lack of contextualization of mhGAP tools and interventions, designed primarily in Western settings, that may not align with the cultural and societal nuances of non-Western or humanitarian settings, potentially undermining local knowledge and practices of care: “*.because these classification systems were created more in the Western world and they do not necessarily apply to these kind of settings.For example, you cannot treat people in the traditional way for PTSD when there is no “post”, they are in a continuous situation. It is a continuous traumatic stress. So not only will the treatment possibly not be effective, but it's also could be a waste of resources, human resources, financial resources. But at the same time, it also undermines the person's experience and it kind of puts the blame on them*” (mental health researcher).

#### 3.3.4 Training relevance

Despite these limitations, the interviewees agreed that *WHO* mhGAP training has been a critical step in equipping primary care professionals with essential skills: “*the assessment, the treatment plan, they gave us clues about how to give the psychoeducation.then exercise deep social relationships and sleep routines. Everything was given in detail, and in every training they repeat the same, important clinical diagnostic criteria.and the treatment plan*” (primary care practitioner, Lebanon).

The modules were considered logical and well structured, and the content was accessible and clear, catering well to primary care staff. Roleplay and practice sessions were particularly favoured because they offered hands-on experience: “*through these courses we were informed about the most common cases which might have appear or come to us. We also studied it from a theoretical point of view and practically because we did the scenarios (roleplay)” (*primary care practitioner, Iraq).

Training sessions conducted outside primary health clinics were appreciated, as they provided a dedicated learning environment. The training also fostered a sense of recognition and validation for the efforts of the primary care practitioners: “*they feel they have something to hold on to, some kind of guideline, something that not only justifies that they spend that time with mental health issues of their patients.but also that they have some ideas as what to do with them. And I think this is one of the main effects of the training*” (mental health researcher).

### 3.4 Perceived mhGAP training impact

When analysing the perceived impact of mhGAP training, the themes can be organized as follows: (1) impact on primary health personnel, (2) impact on patients, and (3) impact on mental health service delivery.

#### 3.4.1 Impact on primary health personnel

When examining the impact of mhGAP training on primary health care personnel, the emerging subthemes are related to (i) perceived technical competencies in mental health, (ii) practices changed after training and (iii) acknowledgement and motivation towards mental health care. The interviewees agreed that one of the first impacts of training is the enhancement of primary care practitioners’ awareness and understanding of mental health disorders. The interviewees mentioned that training has provided practitioners with guidance on early identification and management of the most common mental health issues, enabling them to recognize the physical complaints that may stem from mental health issues: “*there is a lot of benefits for them..especially on the module of the unexplained health complaints..when we conduct the training, you can see the GPs saying “I remember this case. Yeah. I have this one”. They feel relieved during the training.because they have these people who keep visiting them for different unexplained conditions and they do not have the tools or the clinical expertise how to deal with them” (*mhGAP trainer*)*.

The interviewees mentioned that training has emphasized the importance of communication skills, such as active listening and empathy, and the importance of privacy and confidentiality to build an environment where patients feel safe discussing their feelings and concerns: “…*after the training, I have developed skills in regard to how I receive the patient, how I listen to him, how I build trust, how I make him speak what is maybe difficult to speak..(*primary health practitioner, Lebanon*).*

Another critical area of impact mentioned is the ability to assess alarming signs, such as the risk of suicide. Practitioners agreed that now they are more adept at identifying these signs and responding appropriately: “*We used to forget sometimes to ask the patient..So now, after this training, we are able to differentiate all these things. The red flags, everything. The imminent risk. So I do not think I can miss something or do a wrong diagnosis*” (primary health practitioner).

The ability of practitioners to assess and diagnose mental health conditions and to prescribe psychotropics, however, varies significantly across countries due to differing levels of clinical expertise and local regulations concerning the scope of primary health care. In some countries, primary care practitioners are not expected to diagnose mental conditions, and others are not allowed to prescribe psychotropics.

In addition to local specificities and limitations, the interviewees agreed that training has led to a change in attitudes and clinical practices among primary care practitioners. Some interviewees mentioned that this shift represents a move away from outdated perceptions and beliefs towards mental health towards a more inclusive and comprehensive approach to health care: “*before the mental health problem was a secondary problem. For me, the essential was diabetes.also search hypertension. But now we also put the spot over the mental health* (primary health care, Lebanon).

There is also increased motivation and readiness among health staff to better care for patients with mental illness: “*I do think it does scale up the knowledge. It gives them more self-confidence in handling mental health issues, in detecting them and understanding that mental health issues can present in different ways”* (mental health researcher).

#### 3.4.2 Impact on patients

The interviews agreed that mhGAP training has fostered a more accessible, community-centred approach to mental health care and has had effects on patients. One of the most significant impact has been the improvement of access to mental health care for individuals unlikely to seek support from specialized services. Mental health care, which is traditionally centralized in urban and specialized settings, has been largely inaccessible to vast segments of the population, particularly those in rural or underserved areas: “*the benefits are there because otherwise most of them they would not seek mental health services. But if the doctor or the nurse are trained to identify and, at least, to do psychoeducation and treat them, then the access of mental health treatment is increasing*” (mental health supervisor).

The geographic proximity to primary health care providers plays a crucial role in reducing barriers such as distance, cost, and time that often deter people from seeking help: “*the patient will also benefit because they usually find this easier for them to go to the PHC and not to the mental health services. And for many reasons, one of them is the stigma, one of them is the accessibility..* *So after the trainings, yes, if the implementation goes in the right way, the benefit for the patient is very high”*(mhGAP trainer).

The interviews emphasized that another cornerstone is the awareness-raising in mental health provided by trained primary care practitioners. Through interactions with trained primary health practitioners, patients become more informed about their conditions, leading to greater acceptance and willingness to engage in treatment: “*By increasing the number of the trainees or responsible people for mental health, we are also spreading the awareness regarding mental health services in the community” (*Health manager, Iraq).

As patients learn more about their conditions and the available treatments, they become more open to following through with recommendations, contributing to better health outcomes: “*it is very good for the patients and for their families to see the patient return to his natural life, return to sleep, return to eat naturally. The most satisfying comeback is seeing your patients getting better and going back into life again”* (primary care practitioner).

Moreover, the interviewees mentioned that mhGAP training initiatives can contribute to reducing the overall burden of mental health issues at the community level. By equipping primary care practitioners with the skills to identify and manage mental health conditions, the program may enhance early intervention and prevent the escalation of conditions that would require more intensive and specialized care.

#### 3.4.3 Impact on mental health service delivery

The interviewees noted the impact of mhGAP initiatives on mental health service delivery at the primary health level. One of the key impacts of mhGAP training is its role in raising community awareness about mental health issues, helping to demystify mental health conditions and reduce stigma: “*more awareness after the mhgap training..also we track different sides like the community, like mosques, some community leaders. It was part of the mhGAP to increase access to primary health centers, so people are more open to discussing and treating psychological issues”* (Health manager, Iraq). This increased awareness has been pivotal in mobilizing patients, especially those who may not have recognized the mental health dimensions of their suffering to seek care: “*some of the patients when they come and they feel some improvement they bring also some people, relatives or friends. So, a kind of mobilization”* (primary care practitioner, Iraq).

The scaling up of mental health services because of mhGAP training was mentioned by the participants. Pre and post-training surveys have shown a marked increase in the number of patients receiving care for mental health issues at the primary health level, particularly among patients presenting with persistent physical complaints that often mask underlying mental health conditions: “*Some people come, they do not tell us we are depressed. They tell us I have tachycardia, I have chest discomfort, I cannot sleep at night. So when we do the PHQ-9 questionnaire, we know that this patient has depression or is anxious or he has PTSD. So it starts here. It starts with us with the primary health care physicians*” (primary care practitioner, Lebanon).

The interviewees agreed that the effectiveness of mhGAP also depends on post-training follow up and ongoing supervision for primary health practitioners: “*When we started, the doctor would not see any patients with depression. We started with, I do not know, two, three patients. Then she was diagnosing six, seven patients, then ten, twelve patients. And lately, in every supervision, she presents more than 15 patients”* (mhGAP trainer).

Despite this encouraging increase in access to mental health care, the mental health service utilization embodies a complexity that directly impacts access to care, particularly highlighting a paradox where those in dire need, such as individuals who are severely depressed and isolated, may be the least likely to seek help. Interviews agreed on the need to scale up mental health services to a broader audience but also focused on those who are most in need and may remain unreachable due to the barriers imposed by their condition: “*if you're looking at the indicators, possibly you would say, well, we are covering the people who are seeking the support, but you have such big number of people who are not seeking the support..it is kind of paradox, you would have to truly, truly scale up the support system*” (mental health researcher).

### 3.5 Main barriers and challenges to integrating mental health into primary care

The interviewees agreed that integrating mental health at the primary level is a complex process that goes beyond building technical capacity among primary care practitioners and mentioned several barriers faced by primary care practitioners and health managers. Three interrelated thematic domains emerged regarding the main barriers and challenges to integrating mental health into primary care.

#### 3.5.1 Fragmented mental health systems and centralization of care

The main barrier pointed out by the interviewees was the lack of a comprehensive mental health system and community-based services in most contexts, reinforced by a marked centralization of mental health care in psychiatric hospitals. This limitation poses challenges in coordinating a stepped care approach that impacts the continuity and comprehensiveness of care: “the challenge would be from the other side, from the specialized mental health services provider. From my experience, if they are not engaged from the beginning, before even the training, there will be quite resistance to cooperate and support…this revived frustration for the GPs when they try to communicate or to refer and they face this resistance” (mhGAP trainer).

#### 3.5.2 Resource and financial constraints

The expansion of mental health integration into primary care, particularly mhGAP training, is clearly hindered by logistic and financial constraints. The scarcity of mental health services and trained professionals in many contexts also poses a significant challenge to providing quality mental health care and scaling up mhGAP training, especially in underserved areas: “limited resources and investment in mental health is the main barrier..just imagine in these primary health care facilities in Gaza, as I said, if they detected all the people who had a need for being referred to the psychiatric clinic, it is impossible. They have 38 beds for 2 million people” (mental health researcher).

The availability of specific psychotropics and the cost of medical fees, although reduced, still limit access to mental health care for vulnerable populations.

#### 3.5.3 Resistance among primary care staff

One important barrier to implementation is scepticism and resistance among some primary health staff about integrating mental health into primary care: “*For many of the primary health care staff, we still need to show them that is part of their role, but also to help them to find the way and how to do it, how to make it happen. Because again, it's really a challenge, especially in a country that is facing a huge economic crisis*” (mental health supervisor). The interviewees mentioned that this scepticism is often connected to a lack of mental health training during their medical education and is sometimes related to cultural and religious background.

## 4 Discussion

This study examined the perceived effectiveness of the WHO Mental Health Gap Action Programme (mhGAP) from the perspective of primary care practitioners and key stakeholders operating in humanitarian settings characterized by protracted crises and health system fragility, specifically in Lebanon and Iraq. The findings highlight how mhGAP implementation in such contexts is shaped not only by training content and framework, but also by structural constraints, sociocultural determinants, and the availability of functional primary health care services.

### 4.1 The complexity involved in mental health care in humanitarian settings

In line with prevalence estimates of mental disorders in humanitarian contexts (7, 36), the primary care practitioners involved in the study highlighted the symptoms of depression, anxiety and trauma-related stress as the most common mental health conditions encountered in their respective settings. The complexity of care escalates with cases involving suicide risk and the management of substance abuse, which normally require supervisors’ advice and additional skills in coordinating case management with other health and social services and with the patient's family.

The role of socioeconomic factors, such as income, education, and employment status, cannot be overstated. The cycles of prolonged economic crisis are highlighted as strong social determinants, leading to disparities in the availability and quality of mental health services, particularly in conflict affected areas due to the lack of investment from national authorities, alongside security and logistic constraints to enhance service provision, including the mhGAP initiatives. The interviewees agreed that this financial strain, in addition to political instability and memories of war, not only places a direct burden on individuals but also creates an environment conducive to collective heightened stress and anxiety, fomenting a breeding ground for mental health issues (37).

The stigma surrounding mental health within communities (38), and sometimes among health workers, persists as a significant barrier to accessing mental health care and exacerbating the problem. Deep-seated societal misconceptions contribute to individuals’ reluctance to seek help, fearing judgement or social isolation.

Community-level studies in Baghdad have documented persistent stigmatizing attitudes toward mental illness, reinforcing barriers to help-seeking.

### 4.2 The importance of mhGAP and its adapted implementation

The limited scale of mental health services and lack of continuity of care remain major constraints to address the global burden of mental disorders. This is mainly attributed to restricted funding for capacity-building initiatives, the scarcity of specialized mental health services and a limited workforce, and the use of a centralized psychiatric hospital-based model, which reinforces the lack of coordination between local health services (39–41). Local health policies and regulations on the scope of primary health services can also vary significantly across contexts and sometimes impose constraints on the role of primary care practitioners in mental health assessment and case management, including the prescription of essential psychotropics (42).

In this scenario, the mhGAP training model plays an important role. Consistent with the objectives outlined in the mhGAP Intervention Guide and its humanitarian adaptation (12, 43), and supported by systematic reviews of its implementation (14, 15), participants perceived mhGAP as enhancing knowledge, diagnostic skills, and structured clinical reasoning at the primary care level. Similar findings were identified in our recent scoping review of mhGAP effectiveness in fragile settings (19).

However, translating this acquired knowledge into sustained changes in attitudes and clinical practice appears to be considerably more complex. The literature emphasizes that training alone is insufficient without supportive governance, institutional alignment, and structured implementation strategies (13, 39, 44). In our study, the application of mhGAP competencies was strongly shaped by structural determinants, including national mental health legislation and prescribing regulations (30, 42), the degree of integration between primary and specialized services (24, 41), and the availability of referral pathways within fragile health systems (22, 29).

### 4.3 Comparative perspective: Lebanon and Iraq

The implementation framework of mhGAPs varies significantly across the contexts covered by this study and is shaped by local health system structures and existing expertise in mental health. Different pre-training realities also frame the expected outcomes of mhGAP implementation.

In Iraq, where community-based mental health services, specialists, and clinical protocols adapted for primary care practitioners are scarce (26, 28, 29), mhGAP training tends to focus on the essential principles of mental health care. This includes basic clinical skills, early identification of mental disorders, and providing the participants with guidance to conduct awareness-raising activities at the community level. The recommendations from primary care practitioners and supervisors in these settings often emphasize the need for establishing assessment tools and protocols and improving the clinical skills and autonomy of primary care providers in mental health care.

Conversely, in Lebanon, the presence of established governance structures and policy frameworks (21, 22) drives mhGAP training to be part of a broader systemic shift. This can include the implementation of structured clinical protocols and criteria for systematically identifying and referring patients. Training in such contexts prioritizes clinical procedures, patient flow and access to essential psychotropics. Here, the feedback from primary care practitioners and supervisors often calls for greater investment in basic psychosocial support and scalable psychological interventions at the primary care level, as well as initiatives to raise awareness both within health facilities and in the broader community.

### 4.4 The need of a sustained and comprehensive training approach

A common consensus among all mhGAPs included in this study is the emphasis on supervision sessions and post-training monitoring. These are viewed as essential for effectively shifting primary care practitioners’ attitudes and practices towards mental health care. Continuous supervision and post-training follow-up not only reinforce the acquired knowledge but also address the barriers practitioners face in changing their clinical practices. This ongoing support helps in navigating the complexities of integrating mental health care into primary care settings, especially in environments where mental health still carries a stigma, and/or the structural challenges pose challenges to the provision of comprehensive care.

The need for training contextualization is particularly highlighted by some interviewees, especially when it comes to clinical systems of classification (DSM and ICD) in which although they are reliable systems for guiding scientific communication, they may not accurately reflect the lived experiences of patients, especially in conflict-affected contexts (7, 45).

Proper planning prior to mhGAP training initiatives is crucial for their success and sustainability. This process includes a comprehensive review of local laws and regulations ensuring that the training contents align with legal frameworks and operational capabilities. The coordination mechanisms between primary care providers and specialized mental health services must be established to ensure continuity of care and effective referral systems. As a recurring lesson learned, participants emphasize the step-by-step approach of completing one training module at a time, which allows for focused learning and gradual implementation, reducing the risk of information overload and improving retention. The recruitment and training of local trainers are also essential for adapting the mhGAP modules to the cultural and linguistic context, ensuring relevance and effectiveness. A proper implementation plan and pilot training tests are considered essential for identifying potential challenges and refining the mhGAP training approach before broader implementation (13, 44).

### 4.5 Framework to monitor the mhGAP implementation

The development of reliable indicators for monitoring the effectiveness of the training process is indispensable for evaluating impact, guiding improvements, and ensuring that the mhGAP initiative meets its objectives in enhancing mental health care at the primary level. This emphasis on monitoring is consistent with the implementation guidance embedded in the mhGAP intervention guide and its operational manual, which underscore the importance of supervision, service indicators, and system-level evaluation to sustain impact (12, 13). Similarly, recent reviews of mhGAP implementation highlight variability in outcomes across settings and stress the need for context-sensitive monitoring frameworks (14, 15).

A recurrent point mentioned during the study was the satisfaction of patients with the mental health care received at the primary health level. Although a positive indicator, patient satisfaction can reflect not only the quality of care but also perceptions of accessibility, respect, and understanding from healthcare providers—dimensions that are particularly salient in humanitarian contexts where trust in institutions may be fragile (36). While these aspects are very important and high satisfaction levels are encouraging, they must be critically monitored. Instances such as overmedication, while potentially leading to short-term satisfaction due to immediate symptom relief, may underscore the importance of nonpharmacological interventions and continuous monitoring to ensure that care practices are aligned with evidence-based guidelines and ethical standards (46, 47).

### 4.6 The paradox of the health service coverage in the mhGAP implementation

According to the participants, there are a few but promising data related to the impact of training on mental health service delivery at the primary health level. This observation aligns with broader evidence suggesting that mhGAP training can enhance primary care practitioners’ capacity to recognize and manage common mental disorders and contribute to narrowing the mental health treatment gap (18, 45). By strengthening the integration of mental health within primary care, mhGAP operationalizes the principles of task-sharing and stepped care widely advocated in global mental health (12).

However, when considering the challenges raised by local health managers and training supervisors regarding the extension of training initiatives to remote and conflict-affected areas, a significant disparity becomes evident. Populations in these regions remain largely underserved despite overall improvements in trained facilities. This finding resonates with research on health systems in fragile and conflict-affected settings, which demonstrates that service expansion often occurs unevenly, favouring areas with pre-existing infrastructure and institutional stability (48, 49).

This scenario underscores a paradox within global and local efforts to expand access to mental health care: while mhGAP training effectively enhances mental health services within existing primary health care structures, it simultaneously exposes and reinforces the inequities faced by populations in greatest need—those living in areas where primary health care systems are weak, fragmented, or largely unavailable. The challenge remains to extend these training initiatives to all regions ensuring equitable access to mental health care, but also prioritize the needs of vulnerable populations, especially in settings where conflict and instability exacerbate mental health challenges.

### 4.7 Study strengths and limitations

The main strength of this study is the analysis of the real-life implementation of mhGAP initiatives in humanitarian settings in Lebanon and Iraq based on perceptions, beliefs, and recommendations from local actors. This study focused on understanding the effectiveness of the mhGAP-IG by assessing its perceived strength and limitations and the challenges faced by primary health staff and managers in enhancing access to and quality of mental health care at the primary care level.

This study has several limitations. The participants were recruited via purposive sampling, and while following the inclusion criteria and ensuring gender balance and diversity of functional roles, recruitment may inadvertently introduce selection bias, thereby limiting generalizability to other primary care practitioners in Lebanon and Iraq. This study could be enormously beneficial for cross-analysing qualitative approaches with existing health system data that may reflect changes in mental health system utilization patterns after mhGAP training delivery. Unfortunately, these data were not available.

## 5 Conclusion

This study explored stakeholder perspectives on the implementation and perceived effectiveness of mhGAP training in humanitarian contexts marked by protracted instability and fragile health systems. The findings indicate that mhGAP enhances primary care practitioners’ clinical reasoning and confidence, and ability to identify common mental health conditions within routine primary care. Participants also reported improvements in early detection and increased awareness within health facilities.

Beyond training effects, the study highlights that mhGAP implementation is shaped by pre-existing health system configurations and the availability of specialized services. Supervision, structured referral pathways, monitoring mechanisms, and the adaptation of tools to local realities emerged as decisive factors influencing whether newly acquired competencies translate into sustained practice.

The analysis reveals a structural paradox: while mhGAP strengthens mental health service delivery within functioning primary health care facilities, populations in remote or conflict-affected areas—where mental health needs are often greatest—remain underserved when primary care infrastructure is weak or fragmented. Addressing this gap requires moving beyond training alone toward integrated investments in primary health care capacity and equitable access to health services.

Advancing mhGAP implementation in humanitarian settings therefore demands a dual approach: consolidating provider competencies while simultaneously strengthening the health systems that enable equitable, sustainable, and quality mental health care.

## Data Availability

The raw data supporting the conclusions of this article will be made available by the authors, without undue reservation.

## Footnotes

^1^In this study, the term primary care practitioner refers to frontline health professionals (e.g., general practitioners or nurses) who provide direct clinical care at primary health care facilities and are involved in the assessment and management of patients. The term health manager refers to professionals holding administrative or coordination roles at district, regional, or national level (e.g., Ministry of Health), who are responsible for planning and overseeing mental health training initiatives and service organization, but who do not routinely provide direct patient care in primary health settings.
