# Supplementary material for: Stakeholder perspectives on the WHO Mental Health Gap Action Programme Intervention Guide (mhGAP-IG) in humanitarian settings: a qualitative study in Lebanon and Iraq
Source: Front Health Serv. 2026 Mar 17;6:1726804. doi: 10.3389/frhs.2026.1726804 (PMC13036224; doi:10.3389/frhs.2026.1726804)
Supplement: Supplementary file 1 [file Supplementaryfile1.pdf]

Supplementary Material 1. SRQR Checklist

Item	Description according to SRQR	Manuscript location (Section heading)
**S1. Title**	Concise description of the nature and topic of the study; identifying the study as qualitative or indicating the data collection method is recommended	Title
**S2. Abstract**	Summary of key elements of the study, including background, purpose, methods, results, and conclusions	Abstract
**S3. Problem formulation**	Description and significance of the problem/phenomenon studied; review of relevant theory and empirical work	1.Introduction
**S4. Purpose or research question**	Purpose of the study and specific objectives or research questions	1.2 Study objectives
**S5. Qualitative approach and research paradigm**	Qualitative approach and rationale; identifying the research paradigm if appropriate	2.1 Study design
**S6. Researcher characteristics and reflexivity**	Researchers' characteristics that may influence the research, including experience and role	2.1 Study design
**S7. Context**	Setting/site and salient contextual factors with justification	2.1 Study design; 2.2 Sampling strategy
**S8. Sampling strategy**	How and why participants were selected; criteria for deciding when no further sampling was necessary (saturation)	2.2 Sampling strategy
**S9. Ethical issues pertaining to human subjects**	Documentation of ethical approval, consent procedures, and confidentiality measures	2.5 Ethics declarations
**S10. Data collection methods**	Types of data collected and details of data collection procedures	2.4 Data analysis and reporting
**S11. Data collection instruments and technologies**	Description of instruments and devices used; whether instruments changed over time	2.3 Data collection; 2.4 Data analysis and reporting
**S12. Units of study**	Number and relevant characteristics of participants	3.1 Characteristics of the sample

Item	Description according to SRQR	Manuscript location (Section heading)
**S13. Data processing**	Methods for transcription, translation, data management, anonymization, and verification	2.3 Data collection; 2.4 Data analysis and reporting
**S14. Data analysis**	Process by which themes and interpretations were identified, including analytic approach	2.4 Data analysis and reporting
**S15. Techniques to enhance trustworthiness**	Techniques to enhance credibility and trustworthiness (e.g., saturation, team verification)	2.1 Study design
**S16. Synthesis and interpretation**	Main analytic findings, interpretations, and themes	3 Results
**S17. Links to empirical data**	Use of quotes or excerpts to substantiate analytic findings	3 Results
**S18. Integration with prior work, implications, transferability, and contribution**	Integration of findings with existing literature; implications and contribution to the field	4 Discussion
**S19. Limitations**	Trustworthiness and limitations of findings	4.7 Study strengths and limitations
**S20. Conflicts of interest**	Potential sources of influence or perceived influence and how these were managed	No conflict-of-interest statement
**S21. Funding**	Sources of funding and role of funders in the research	Funding Declaration
